# The Inverse F-BAR Domain Protein srGAP2 Acts through srGAP3 to Modulate Neuronal Differentiation and Neurite Outgrowth of Mouse Neuroblastoma Cells

**DOI:** 10.1371/journal.pone.0057865

**Published:** 2013-03-07

**Authors:** Yue Ma, Ya-Jing Mi, Yun-Kai Dai, Hua-Lin Fu, Da-Xiang Cui, Wei-Lin Jin

**Affiliations:** 1 School of Life Sciences and Biotechnology, Shanghai Jiao Tong University, Shanghai, People's Republic of China; 2 Department of Bio-Nano-Science and Engineering, Institute of Micro-Nano Science and Technology, Shanghai Jiao Tong University, Shanghai, People's Republic of China; 3 Lab of Cell Biology & Translational Medicine, Xi'an Medical University, Xi'an, People's Republic of China; Cleveland Clinic Foundation, United States of America

## Abstract

The inverse F-BAR (IF-BAR) domain proteins srGAP1, srGAP2 and srGAP3 are implicated in neuronal development and may be linked to mental retardation, schizophrenia and seizure. A partially overlapping expression pattern and highly similar protein structures indicate a functional redundancy of srGAPs in neuronal development. Our previous study suggests that srGAP3 negatively regulates neuronal differentiation in a Rac1-dependent manner in mouse Neuro2a cells. Here we show that exogenously expressed srGAP1 and srGAP2 are sufficient to inhibit valporic acid (VPA)-induced neurite initiation and growth in the mouse Neuro2a cells. While ectopic- or over-expression of RhoGAP-defective mutants, srGAP1^R542A^ and srGAP2^R527A^ exert a visible inhibitory effect on neuronal differentiation. Unexpectedly, knockdown of endogenous srGAP2 fails to facilitate the neuronal differentiation induced by VPA, but promotes neurite outgrowth of differentiated cells. All three IF-BAR domains from srGAP1-3 can induce filopodia formation in Neuro2a, but the isolated IF-BAR domain from srGAP2, not from srGAP1 and srGAP3, can promote VPA-induced neurite initiation and neuronal differentiation. We identify biochemical and functional interactions of the three srGAPs family members. We propose that srGAP3-Rac1 signaling may be required for the effect of srGAP1 and srGAP2 on attenuating neuronal differentiation. Furthermore, inhibition of Slit-Robo interaction can phenocopy a loss-of-function of srGAP3, indicating that srGAP3 may be dedicated to the Slit-Robo pathway. Our results demonstrate the interplay between srGAP1, srGAP2 and srGAP3 regulates neuronal differentiation and neurite outgrowth. These findings may provide us new insights into the possible roles of srGAPs in neuronal development and a potential mechanism for neurodevelopmental diseases.

## Introduction

The Slit-Robo GTPase-activating proteins (srGAPs) were originally identified as a downstream mediator of neuronal repellent factor Slit and Robo receptor [1]. In mammals, the srGAP family consists of four members, srGAP1, srGAP2, srGAP3 and distantly related srGAP4 (also known as ARHGAP4/RhoGAP C1) [2]. The srGAPs proteins share considerable structural and functional homology. They all possess three functional domains: an N-terminal FCH-Bin/Amphiphysin/Rvs (F-BAR) domain, a central RhoGAP domain, and C-terminal tail containing a Src homology 3 (SH3) domain [2], [3]. Functionally, this family of Rho-GAPs collectively defines an “inverse F-BAR” or IF-BAR domain that is distinct from other F-BAR domains such as FBP17 [4].

Accumulating data suggest that the srGAP1, 2 and 3 proteins are important multifunctional adaptor proteins involved in various aspects of neuronal development, including axon guidance, neuronal migration, neurite outgrowth, dendritic morphology, spine maturation and synaptic plasticity [1], [3]–[8]. Partially overlapping expression pattern [9], [10] and highly homologous protein structures indicate that srGAPs may play distinct and redundant roles in neuronal development. For example, several investigations demonstrate three srGAPs negatively regulate neuronal migration [1], [3] and axon guidance [7]. SrGAP1, the prototype of the srGAP family, modulates Slit-Robo-dependent repulsive cues and migration of anterior subventricular zone (SVZa) neurons by inactivating the small Rho GTPase Cdc42 and inhibiting actin polymerization [1]. SrGAP2 negatively regulates cortical neuronal migration through the ability of its IF-BAR domain to induce filopodia-like membrane protrusions [3]. SrGAP3 may play an important role in the lateral positioning of post crossing axons within the ventrolateral funiculus of mouse spinal cord, possibly downstream of Robo1 [7].

Other investigations demonstrate that srGAP2 and srGAP3 elicit opposite effects on neurite outgrowth [3], [5] and dendritic spine formation [4], [8]. Different from srGAP2 promoting neurite outgrowth and branching through its IF-BAR domain [3], srGAP3/WRP has been shown to regulate Rac1 and Cdc42 and inhibit Rac1-dependent neurite outgrowth [5]. Very recently, more detailed data from srGAP2 and srGAP3 gene knockout mice suggest a distinct regulatory role in spine maturation [4], [8]. Loss of srGAP3 *in vivo* and *in vitro* results in reduced density of spines [4]. *In vivo* loss of srGAP3 causes a loss of mushroom-shaped spines. Meanwhile, srGAP2-deficient neurons harbored spines with longer necks and higher spine density [8].

It has been shown that SRGAP genes may be linked to some neurodevelopmental disorders such as mental retardation, schizophrenia and seizure. SRGAP3, alternate name of Mental-Disorder Associated GAP Protein (MEGAP) is reported to be disrupted and functionally inactivated by a translocation breakpoint in a patient with a severe form of mental retardation, the *3p-* syndrome [11]. Case report identified the first family of a SRGAP3 copy number variant (CNV) in schizophrenia [12], [13]. SRGAP3 knockout mice lead to lethal hydrocephalus or ‘schizophrenia-related’ behaviors [4], [14], [15]. SRGAP2 has also recently been implicated in a severe neurodevelopmental syndrome causing early infantile epileptic encephalopathy [16] and SRGAP2 knockout mice are prone to epileptic seizures [8]. The molecular mechanisms underlying neuronal development and diseases remain to be clarified.

Besides inducing plasma membrane deformation, the srGAPs negatively regulate Rho family GTPase activity, and therefore modulate signaling events that control cytoskeletal dynamics, they also play diverse roles in neuronal differentiation of neuroblastoma (NB) cell lines [17]–[20], and also in cell spreading and migration in non-neuronal cells [21]–[23]. For instance, in mouse NIE-115 cells, knockdown of srGAP2 increases the number of filopodia and initials cell spreading [19] and silencing of srGAP3 facilitates the formation of neurite-like processes [17]. Knockdown of endogenous srGAP3 in mouse Neuro2a cells also facilitates the VPA-induced neuronal differentiation [20]. Transfection of the srGAP3 gene into human SHSY-5Y that lack detectable srGAP3 protein has been shown to reduce cell migration and protrusion formation as a result of downregulation of Rac1 signaling [18]. The same group recently reported that srGAP3 interacts with lamellipodin and has an inhibitory role on actin dynamics, specifically on lamellipodia formation [17].

Very recently, three srGAP family members were identified to form both homo- and hetero-dimers through IF-BAR domain [3], [24]. In the present study, we report that the synergistic interactions of srGAP1, srGAP2 and srGAP3 negatively regulate neuronal differentiation and neurite outgrowth of mouse Neuro2a cells.

## Results

### SrGAPs inhibit neuronal differentiation and neurite outgrowth of VPA-induced Neuro2a cells

**Figure 1:**
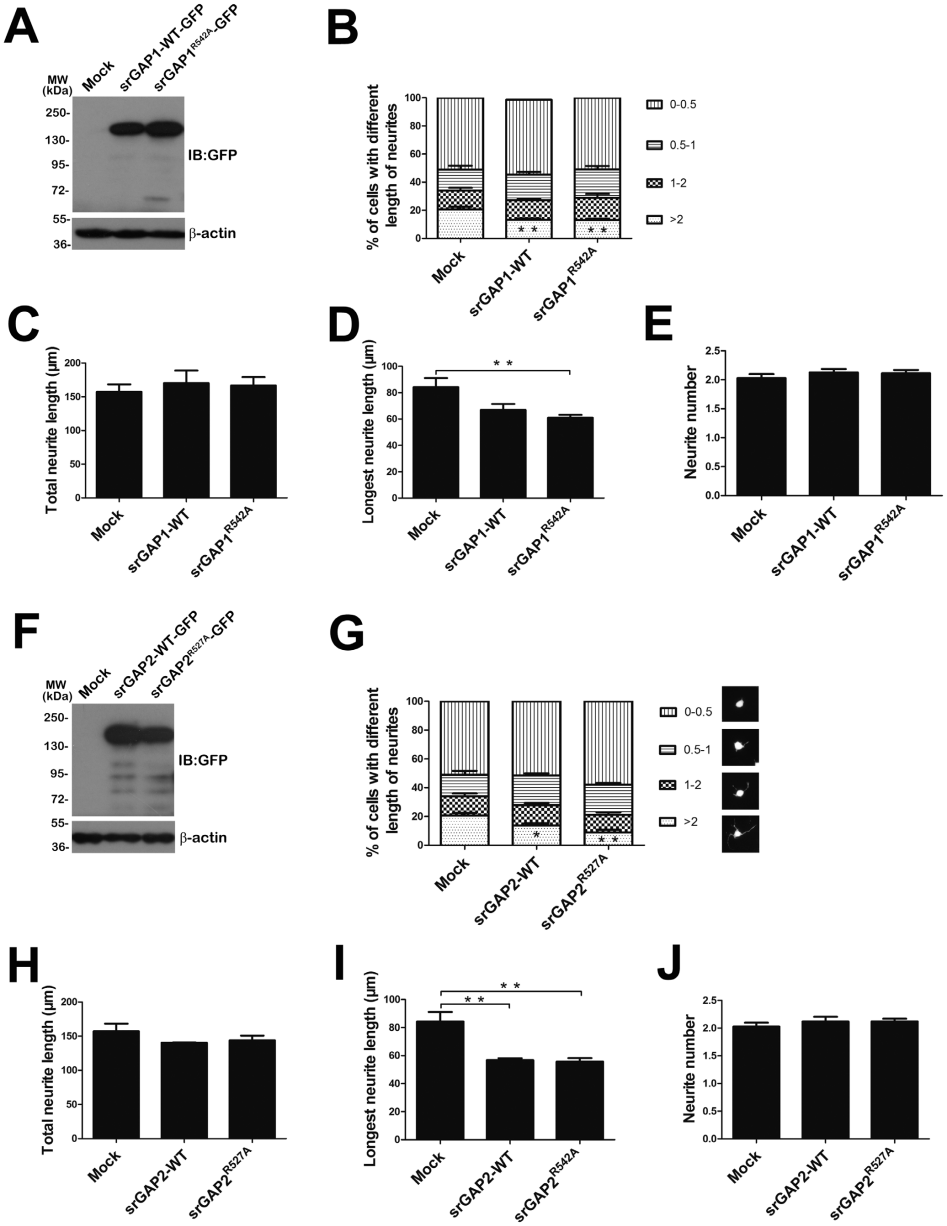
SrGAP1 and srGAP2 inhibit neuronal differentiation and neurite outgrowth of VPA-induced Neuro2a cells. A and F. The proper expression of srGAP1-WT/srGAP1^R542A^ and srGAP2-WT/srGAP2^R527A^ was confirmed by Western blot with GFP antibody. β-actin was selected as a loading control. B and G. Analysis of cell percentage of VPA-induced Neuro2a cells sharing different length of neurites. *n* = 3, Mean ± S.D., one-way ANOVA, **P<0.01. C–E and H–J. Assessment of neurite outgrowth of Neuro2a cells by VPA stimulation for 24 h. Three endpoints were quantified: total neurite length (C and H), longest neurite length (D and I), and neurite number per cell (E and J). C. Total neurites length of srGAP1 or srGAP1^R542A^ over-expression (srGAP1-WT, 175.40±17.99 µm; srGAP1^R542A^, 156.10±13.84 µm; mock, 162.90±7.87 µm). D. Longest neurite length of srGAP1 or srGAP1^R542A^ over-expression (srGAP1-WT, 66.98±4.52 µm; srGAP1^R542A^, 61.10±2.17 µm; mock, 84.25±6.88 µm). E. Neurite number of srGAP1 or srGAP1^R542A^ over-expression (srGAP1-WT, 2.128±0.059; srGAP1^R542A^, 2.114±0.056; mock, 2.028±0.071). H. Total neurites length of srGAP2 or srGAP2^R527A^ over-expression (srGAP2-WT, 159.00±11.20 µm; srGAP2^R527A^, 138.90±3.27 µm; mock, 143.70±2.11 µm). I. Longest neurite length of srGAP2 or srGAP2^R527A^ over-expression (srGAP2-WT, 56.67±1.45 µm; srGAP2^R527A^, 55.73±2.44 µm; mock, 84.25±6.88 µm). J. Neurite number of srGAP2 or srGAP2^R527A^ over-expression (srGAP2-WT, 2.120±0.086; srGAP2^R527A^, 2.122±0.048; mock, 2.028±0.071). *n* = 3, Mean ± S.D., one-way ANOVA, *P<0.05; **P<0.01.

We found previously that srGAP3 can inhibit VPA-induced neuronal differentiation in Neuro2a cells by a Rac1-dependent manner [20]. To address directly the question of functional complementation among srGAP family members, we extended to evaluate their roles of srGAP1 and srGAP2 in neuronal differentiation and neurite outgrowth. Neuro2a cells were transiently transfected with an EGFP-tagged srGAP1-WT, srGAP2-WT and their RhoGAP domain mutant forms of srGAP1^R542A^ or srGAP2^R527A^ expression vectors. The single conserved arginine residue in the RhoGAP domain from srGAP2 and srGAP3 has been proposed to be involved in catalysis (Fig. S1C; [3], [18], [20]). The transfected cells were labeled by GFP. Western blots have demonstrated that srGAP1 and srGAP2 were properly expressed in Neuro2a cells (Fig. 1A and F). The effects of srGAP1, srGAP2 and their RhoGAP mutants on VPA-induced Neuro2a cells neurite outgrowth and differentiation were analyzed. After treatment of VPA for 24 h, the percentages of neurite-bearing cells in four categories were quantified (as shown in Fig. 1B and G). Cells with neurite processes longer than two cell bodies were considered to be differentiated. Over-expression of srGAP1, srGAP2 and their RhoGAP mutants led to a significant inhibitory effect on neuronal differentiation by VPA stimulation. Over-expression of srGAP1-WT or srGAP2-WT in the Neuro2a cells had an approximately 40% inhibitory effect on neuronal differentiation by VPA stimulation for 24 h (Fig. 1B and G). Compared to mock transfection, which was 20.70±1.80%, the differentiation rate was reduced to 13.29±1.00% (srGAP1-WT, Fig. 1B), and 13.75±1.59% (srGAP2-WT, Fig. 1G), respectively. While over-expression of srGAP1^R542A^ or srGAP2^R527A^ also resulted in a remarkable reduction in differentiation rates, from 20.70±1.80% to13.06±1.07% (srGAP1^R542A^, Fig. 1B) or 8.86±1.94% (srGAP2^R527A^, Fig. 1G).

We next assessed whether srGAP1 and srGAP2 could affect neurite outgrowth of differentiated Neuro2a cells by quantified total neurite length, longest neurite length and neurite number per cell. The results showed that over-expression of srGAP1, srGAP2 and their GAP-dead mutants did not affect total neurite length (Fig. 1C and H) or the average neurite number (Fig. 1E and J), but remarkably decreased longest neurite length (Fig. 1D and I).

Our results clearly showed that ectopic- or over-expression of srGAP1, srGAP2 and also their GAP-dead mutants inhibit neuronal differentiation and neurite outgrowth of differentiated Neuro2a cells independent of their RhoGAP activity, which is very different from the effect of srGAP3 and srGAP3^R542A^ on neuronal differentiation [20].

### Knockdown of endogenous srGAP2 fails to facilitate neuronal differentiation of Neuro2a cells

**Figure 2:**
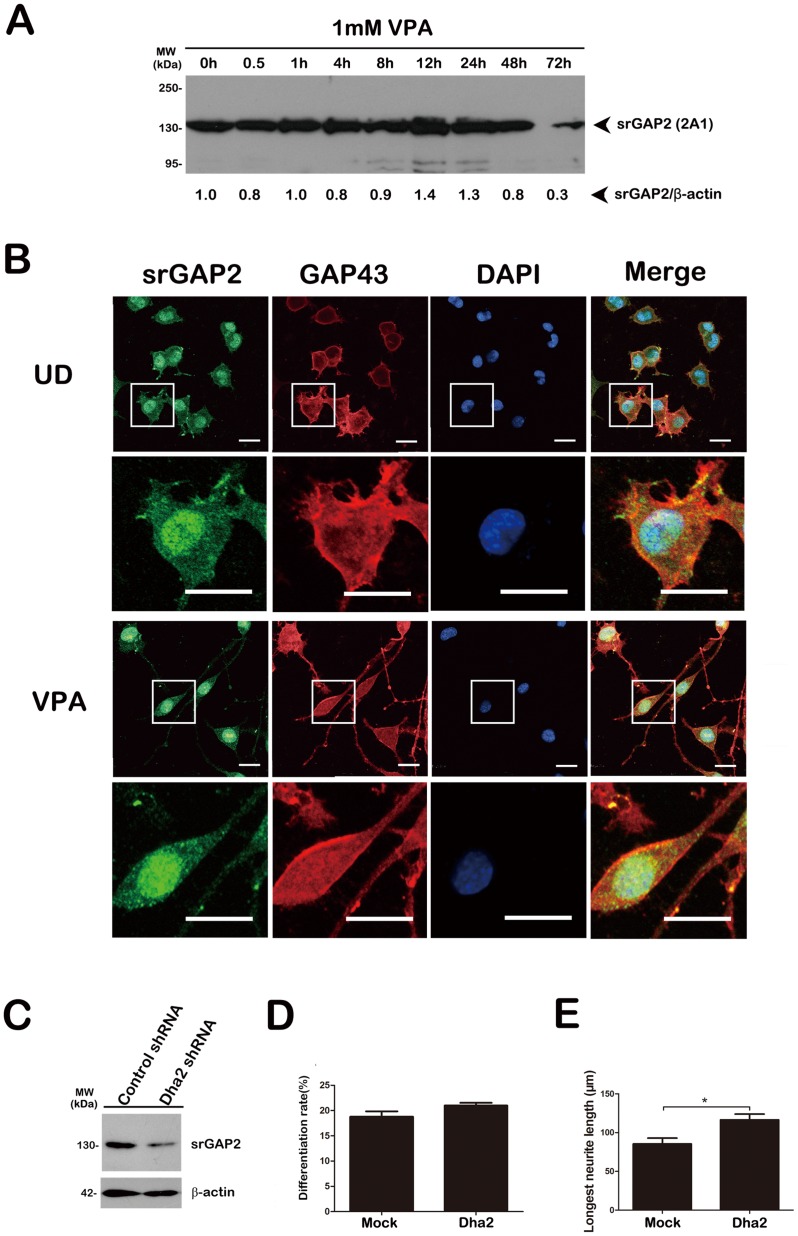
Knockdown of endogenous srGAP2 fails to facilitate neuronal differentiation of Neuro2a cells. A. Lysates from Neuro2a cells exposed to VPA for the indicated times were subjected to Western blot analysis with srGAP2 (2A1) antibody. The relative expression level of srGAP2 was calculated by srGAP2/β-actin. B. Neuro2a cells exposed to VPA or not (UD) were co-immunostained with srGAP2 (2A1) and GAP-43 antibodies. The lower panels show magnifications of the single cell and process outlined by the white box in the upper panels respectively. Bar = 50 µm. C. The knockdown efficiency of Dha2 shRNA against srGAP2 in Neuro2a cells was confirmed by Western blot. β-actin is selected as a loading control. D and E. The effect of srGAP2 knockdown on Neuro2a cells differentiation rate (D) and the longest neurites length (E). *n* = 3, Mean ± S.D., paired *t*-test, *P<0.05.

Our RT-PCR analysis had demonstrated the presence of mRNAs of srGAP2 and srGAP3, not srGAP1 in the Neuro2a cells [20]. Our Western blot analysis had shown that the expression of endogenous srGAP3 level in Neuro2a cells was up-regulated during early differentiation phase and srGAP3 was down-regulated when the neuronal differentiation reaches maturation stage [20]. The similar expression pattern of srGAP2 was also observed in the blot (Fig. 2A). Immunofluorescence staining of un-differentiated (UD) and differentiated (VPA) Neuro2a cells demonstrated that endogenously expressed srGAP2 also localized to the cytoplasm, the nucleus, filopodia and lamellipodia structures at the cell periphery of Neuro2a cells (Fig. 2B).

To exploit if endogenous srGAP2 is involved in neuronal differentiation, we knockdown its expression by two shRNA constructs of Dha2 [3] and J24 (see Fig. S2). We transfected Dha2 construct with an empty pEGFP-N1 vector to visualize transfected cells. Two shRNAs could effectively knockdown exogenously expressed srGAP2 in HEK293T cells (see Fig. S2A) [3] and Neuro2a cells (Fig. 2C and Fig. S2B). Under the above inducing conditions, we did not observe a significant increase in neurite-bearing cells in srGAP2 (Dha2 and J24)-knockdown cells compared with shRNA controls (control shRNA: 18.79±1.06%; Dha2 shRNA: 20.09±0.55%) (Fig. 2D), but we observed a remarkable increase of the longest neurite length of srGAP2 (Dha2)-knockdown cells from 85.45±7.45 µm to 116.60±7.35 µm (Fig. 2E). Combined with the endogenous expression patterns of srGAP2 down-regulated after the treatment of VPA for 72 h (Fig. 2A), these data suggest that srGAP2 is redundant for neuronal differentiation, but is required for neurite outgrowth.

### Hetero-interaction of srGAP2 and srGAP3 in Neuro2a cells

Based on their high degree of homology (Fig. S1A), and the ability of IF-BAR domains of srGAP1-3 forming homo- and hetero-dimers [3] [24], we wanted to test the redundant and synergistic mechanisms of srGAP1, srGAP2 and srGAP3 regulating neuronal differentiation in Neuro2a cells. We firstly confirmed that all three IF-BAR domains are structurally conserved and are capable of hetero-dimerization or oligomerization in HEK293T cells [24].

**Figure 3:**
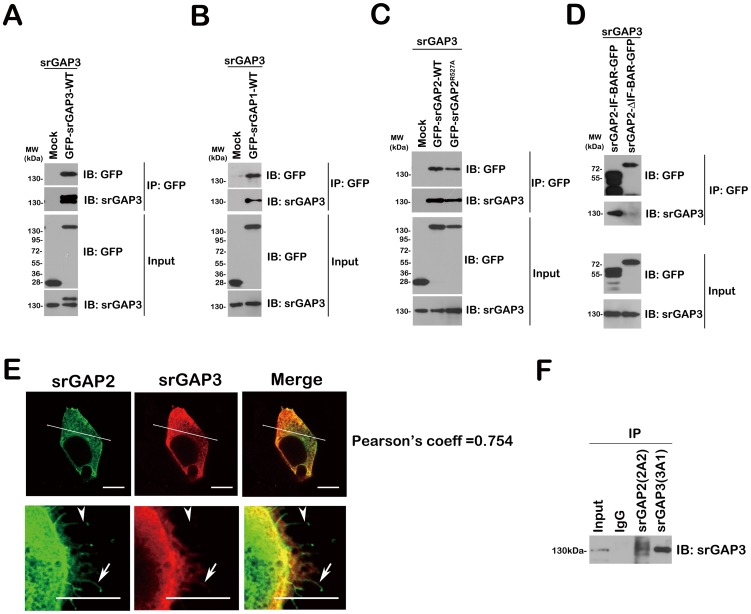
The interactions of the three srGAPs. A–D. Non-tagged srGAP3 and GFP-tagged wild/mutated srGAPs or deletions were co-transfected into HEK293FT, and immunoprecipitated with GFP antibody. (A) Lane1: Mock; lane2: srGAP3-WT. (B) Lane1: Mock; lane2: srGAP1-WT. (C) Lane1: Mock; lane2: srGAP2-WT; lane3: srGAP2^R527A^. (D) Lane1: srGAP2-IF-BAR; lane2: srGAP2-ΔIF-BAR. E. Non-tagged srGAP3 and GFP-tagged wild type srGAP2 were co-transfected into HEK293FT, and then immunostained with srGAP3 antibody (3A1). The pearson's coefficient of Correlation (short for r) is a measure of the degree of colocalization of srGAP2 and srGAP3. The left panels, upper: one type cell with no filopodia; lower: the other type cell with numerous filopodia. Arrowhead: srGAP2 (+) and srGAP3 (−); arrow: srGAP2 (+) and srGAP3 (+). Bar = 5 µm. F. Neuro2a lysates were immunoprecipitated with srGAP2 (2A2) and srGAP3 (3A1) antibodies, and then blotted with another srGAP3 antibody, 3A3.

Combinations of non-tagged srGAP3 and GFP-tagged srGAPs were co-transfected into HEK293FT cells and immunoprecipitated with a GFP antibody (Fig. 3A–D). Western blots were probed for 3A1, revealing interactions between srGAP3 and all three full-length srGAP proteins (Fig. 3A–C). Meanwhile, GFP-tagged srGAP2 IF-BAR, not GFP-tagged srGAP2-(IF-BAR immunoprecipitated srGAP3 indicates that this interaction occurred through the respective IF-BAR domains, and not through indirect interaction through SH3 domain binding (Fig. 3D). Then, we examined for co-localization of the proteins in HEK293FT cells, by cotransfection of non-tagged srGAP3 and GFP-tagged srGAP2 and immunofluorescence staining with 3A1 antibody (Fig. 3E). In two examples of specialized cells, we clearly observed co-localization of GFP-srGAP2 and srGAP3 proteins, mainly in punctate cytoplasmic structures, plasma membrane and some protrusions (Fig. 3E). We also noticed that when co-expressed, srGAP2 and srGAP3 showed distinct distribution along the filopodia (Fig. 3E, lower panel).

We then detected in vivo interaction of the endogenous co-expression of srGAP2 and srGAP3 in mouse Neuro2a cells by immunoprecipitation with srGAP2-2A2 and srGAP3-3A1 (Fig. 3F). Results from immunoprecipitation studies and Western blot with another srGAP3 antibody, 3A3 indicate that srGAP2 interacts to srGAP3.

### SrGAP3 is required for srGAP2 over-expression induced neuronal differentiation inhibition

**Figure 4:**
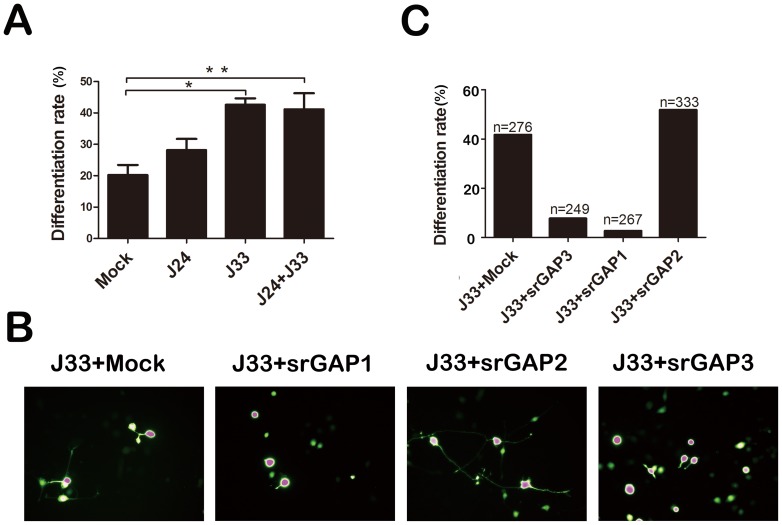
SrGAP3 is required for srGAP2 over-expression induced neuronal differentiation inhibition. A. J24 shRNA against srGAP2 and J33 shRNA against srGAP3 were transfected into Neuro2a cells alone or combined. Then cell differentiation rate is analyzed. *n* = 3, Mean ± S.D., one-way ANOVA, *P<0.05; **P<0.01. B. The three srGAP1-3 over-expression constructs were respectively transfected into the srGAP3-depleted Neuro2a cells with J33 shRNA. C. Cell differentiation rate in (B) is analyzed.

As some biochemical evidence had shown that srGAP2 can interact with srGAP3 (Fig. 3C–G), we wanted to test if srGAP3 could be involved in srGAP2 over-expression induced neuronal differentiation inhibition. We firstly quantified the number of neurite-bearing Neuro2a cells co-transfected with the knockdown constructs for srGAP2 (J24) or srGAP3 (J33) alone, or in combination. Whereas knockdown of srGAP2 (J24) alone did not have a statistically significant effect (control shRNA: 20.25±3.17; J24 shRNA: 28.16±3.57), we found that knockdown of srGAP3 or double knockdown efficiently inhibit VPA-induced neuronal differentiation (J33 shRNA: 42.60±2.07; J24+J33 shRNA: 41.15±5.11) (Fig. 4A). We then quantified the number of neurite-bearing Neuro2a cells co-transfected with the knockdown construct for srGAP3 and full-length srGAPs expression vectors. As expected, over-expression of srGAP2 did not show any inhibitory effect (Fig. 4B and C), but both srGAP1 and srGAP3 efficiently inhibit VPA-induced neuronal differentiation of srGAP3-depleted Neuro2a cells (Fig. 4B and C). These results indicate that srGAP3 may mediate srGAP2 over-expression induced neuronal differentiation inhibition.

### Endogenous srGAP2 has a weak RhoGAP activity towards Rac1

Our previous study had confirmed that Rac1 signaling mediated srGAP3 inhibiting neuronal differentiation in Neuro2a cells [20]. It has reported that srGAPs have different preferred substrates of Rho GTPases in vitro [1], [3], [5], [11], [18], [23]. However, the in vivo functions of GAPs are not always identical to their GAP activities in vitro [1], [5], [18].

**Figure 5:**
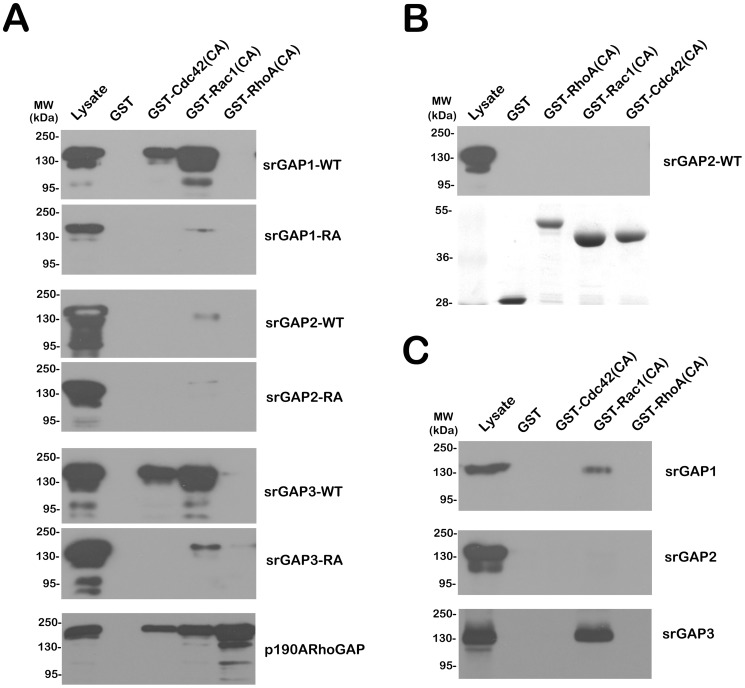
Endogenous srGAP2 has a weak RhoGAP activity towards Rac1. A. RhoGAP pull-down assay by GST-CA Cdc42, Rac1 and RhoA was used to precipitate three GFP-tagged srGAPs (WT) and their RhoGAP mutants (RA) in transfected HEK293T cells. P190A RhoGAP was selected as a positive control. B. RhoGAP pull-down assay was performed to precipitate endogenous srGAP2 in Neuro2a cells. The amount of GST-fusion protein used in the assay was revealed by naphthol blue black staining (lower). C. RhoGAP pull-down assay was performed to precipitate endogenous srGAP1-3 from P15 rat cerebral cortex.

The RhoGAP pull-down assay [20], [25] was firstly used to precipitate three GFP-tagged srGAPs and their RhoGAP mutants in transfected HEK293T cells. Full length GFP-tagged srGAP1 and srGAP3 strongly interacted with GST-CA Rac1 and Cdc42, while the “GAP-dead” srGAP1R542A and srGAP3R542A only weakly interacted with GST-CA Rac1 (Fig. 5A). Unexpectedly, both full length GFP-tagged srGAP2 and its RhoGAP mutant interacted very weakly with GST-CA Rac1. It also indicates that srGAP2 may have an indirect binding ability toward Rac1.

We extended the RhoGAP pull-down assay by GST-CA Cdc42, Rac1 and RhoA in Neuro2a cells (Fig. 5B) and rat P15 cortical lysates (Fig. 5C). We observed that only GST-CA Rac1 can precipitate endogenous srGAP1 and srGAP3. Consistent with the data from HEK293T, endogenous srGAP2 from Neuro2a cells (Fig. 5B) and P15 cortex (Fig. 5C, middle panel) do not bind to CA-Cdc42, Rac1 or RhoA. These data suggest that Rac1 could serve as a direct downstream effector for both srGAP1 and srGAP3, not srGAP2.

### The isolated IF-BAR domain from srGAPs induces filopodium formation

Recently, the IF-BAR domain presented in srGAPs have been reported to induce filopodia in COS7 cells [3], [4], [24] and cortical neurons, resulting in neurite outgrowth, branching, spine formation and maturation.

**Figure 6:**
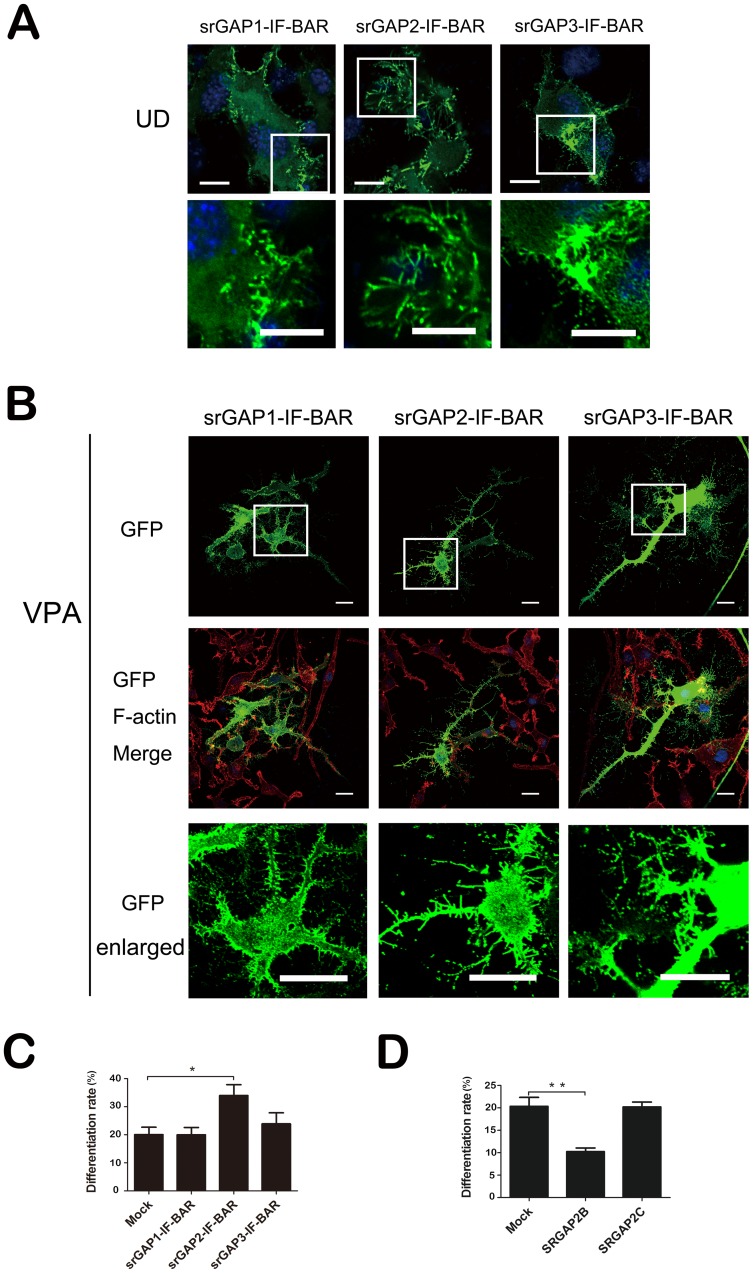
IF-BAR domains from srGAPs induce filopodia formation. A. Undifferentiated Neuro2a cells (UD) were over-expressed by srGAP1-3 IF-BARs. Bar = 20 µm. The lower panels show magnifications of the filopodia and protrusions outlined by the white box in the upper panels respectively. Bar = 10 µm. B. VPA-induced Neuro2a cells over-expressed by GFP-tagged srGAP1-3 IF-BARs were stained with Texas Red-X phalloidin. Bar = 20 µm. The lowest panels show magnifications of the filopodia and protrusions outlined by the white box in the upper panels respectively. Bar = 10 µm. C. Analysis of cell differentiation rate of VPA-induced Neuro2a cells over-expressed by the three IF-BARs. *n* = 3, Mean ± S.D., one-way ANOVA, *P<0.05. D. The effect of SRGAP2B and SRGAP2C on Neuro2a cells differentiation rate. *n* = 3, Mean ± S.D., paired *t*-test, *P<0.05.

To examine if the IF-BAR domain from srGAPs is responsible for srGAPs over-expression inhibiting neuronal differentiation, we transfected plasmids expressing isolated IF-BAR domains from three srGAPs fused in their C-terminal end to enhanced green fluorescent protein (EGFP) into Neuro2a cells. GFP-tagged srGAP1-IF-BAR, srGAP2-IF-BAR and srGAP3-IF-BAR induce more filopodia and numerous small protrusions in the neurites than EGFP alone (unpublished data) in the absence (Fig. 6A) or presence of VPA (Fig. 6B). Notably, these protrusions, particularly when induced by the IF-BAR domain of srGAP1-3, differ from canonical filopodia since they generally display a lower content of organized F-actin. We didn't observe that srGAP2-IF-BAR domain has significantly more potent filopodia inducing ability than srGAP1-IF-BAR and srGAP3-IF-BAR in Neuro2a cells, like in COS7 cells [8], [24].

We quantified the number of neurite-bearing Neuro2a cells transfected with the constructs for each respective IF-BAR domain. Whereas over-expression of srGAP1-IF-BAR and srGAP3-IF-BAR did not have a statistically significant effect, we found that srGAP2-IF-BAR over-expression efficiently promotes VPA-induced neuronal differentiation from 20.06±2.62% to 33.98±3.86% (Fig. 6C). We also noticed srGAP3-IF-BAR slightly increases neuronal differentiation (Fig. 6C).

Although SRGAP2B and SRGAP2C, products of two human duplications of SRGAP2 gene 8,26 retained the capacity to bind to negatively charged lipid (Figure S3A), they lost the ability to induce filopodia in COS7 (8), HEK293T (Figure S3B) and Neuro2a cells (Fig. S3C). Furthermore, we quantified the number of neurite-bearing Neuro2a cells transfected with the constructs for SRGAP2B and SRGAP2C. Unlike the phenotype of srGAP2-IF-BAR, we found that over-expression of SRGAP2B, not SRGAP2C efficiently inhibits VPA-induced neuronal differentiation from 20.36±1.98% to 10.26±0.81% (Fig. 6D).

The findings that the isolated IF-BAR domain can induce filopodium formation and not inhibit neuronal differentiation, indicate that the inhibitory effect of srGAPs may be independent of filopodia inducing activity of their IF-BAR domain.

### Disturbance of Slit-Robo signaling enhances neuronal differentiation of Neuro2a cells

**Figure 7:**
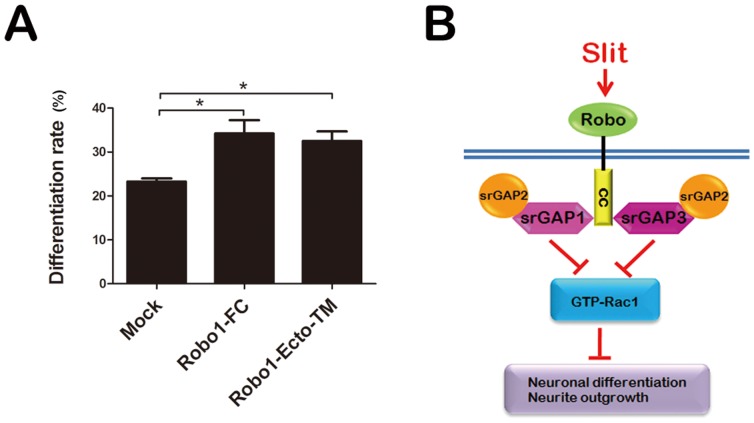
Slit-Robo signaling involves in neuronal differentiation of Neuro2a cells. A. Robo1-Fc or Robo1ecto-TM plasmids were transfected into Neuro2a cells, and cell differentiation rate was analyzed. *n* = 3, Mean ± S.D., one-way ANOVA, *P<0.05. B. A working model proposed that srGAPs proteins may regulate NB cell differentiation as a mediator of Slit2-Robo1 signaling pathway. The IF-BAR domain might localize srGAPs to the plasma membrane, where they interact with Robo1 through their SH3 domain. Slit2 binds to Robo1 receptor and subsequently activates srGAP3 RhoGAP activity. Downregulation of GTP-bound Rac1 activity leads to inhibition of neuronal differentiation in Neuro2a cells.

Slit-Robo signaling plays important roles in the axon guidance, axon branching, neuronal migration and morphological differentiation [1], [27]. Very recently, Huang et al reported that Slit2 inhibits cell motility and neuronal differentiation of SHSY-5Y cells [28], and Sung et al., identified ROBO2 gene involvement in neuroblastoma cell differentiation by a microarray analysis [29]. More importantly, Slit2, Robo1 and Robo2 are found to be highly expressed in Neuro2a cells [20], [30]. We employed two antagonists, Robo1-Fc fusion protein and a dominant-negative Robo, Robo1ecto-TM to inhibit Slit-Robo interactions [31], [32]. We quantified the number of neurite-bearing Neuro2a cells transfected with the Robo1-Fc or Robo1 ecto-TM constructs. We found that both have a statistically significant positive effect of neuronal differentiation (mock transfection: 23.28±0.70%; Robo1-Fc over-expression: 34.22±3.03%; Robo1ecto-TM over-expression: 32.50±2.717%) (Fig. 7A), which is similar to the phenotype when srGAP3 is inhibited (Fig. 4A). These data raise the intriguing possibility that srGAPs proteins could actually regulate NB cell differentiation acting as a mediator of Slit2-Robo1 pathway.

## Discussion

In this report, we have shown that overexpression of srGAP1 and srGAP2 inhibits VPA-induced neurite outgrowth in Neuro2a cells in a GAP-independent manner. We demonstrate that the three srGAP family members can form both homo- and hetero-dimers through IF-BAR domain. Functionally, srGAP1, srGAP2 and srGAP3 cooperate to act as a negative inhibitor of neuronal differentiation in the downstream of Slit-Robo pathway (Fig. 7B). In addition of srGAP2 inhibitory function through interacting with srGAP3, we now report on a distinct role of srGAP1 and srGAP2 in neurite outgrowth, rather than srGAP3. Furthermore, we show that SRGAP2B, protein product from one copy of human duplications of SRGAP2 gene, has the opposite effect of the isolated srGAP2 IF-BAR domain, inhibiting VPA-induced neuronal differentiation of Neuro2a cells.

Extensive studies suggest distinct and overlapping functions of srGAPs in neuronal development [1], [3]–[8]. We assessed the redundant and synergistic interaction of srGAPs proteins in VPA-induced Neuro2a cell model [20]. Although over-expression of three srGAPs inhibits neuronal differentiation (Fig. 1), the effect of srGAP2 is biochemically and functionally dependent on srGAP3 (Fig. 3 and 4). Very interestingly, srGAP1 and srGAP2, not srGAP3 inhibit neurite outgrowth of differentiated cells (Fig. 1; [20]). We proposed that the distinct and overlapping functions between srGAPs may be due to the functional diversity of the membrane deformation properties of this subclass of IF-BAR-domains, the preferred substrates of RhoGAP domains and binding partners of SH3 domains.

Three srGAPs share highly homologous protein structural features [3]. Specially, the IF-BAR domains share approximately 85% amino-acid identity. On the biological level, all three IF-BAR domains can induce filopodium formation [3], [4]. On the molecular level, these three IF-BAR domains can heterodimerize and act synergistically towards filopodia induction [24]. In COS7 cells, srGAP2-IF-BAR displays faster molecular dynamics than srGAP3-IF-BAR and srGAP1-IF-BAR at the plasma membrane which correlates well with its increased potency to induce filopodia. Consistent with the various membrane deformation properties [24], three isolated IF-BAR domains have different activity in filopodia formation and neurite initiation. Different from full-length or “GAP-dead” srGAP2 overexpression inhibiting VPA-induced neuronal differentiation in Neuro2a cells, srGAP2 IF-BAR not only induces filopodia, but also promotes neurite initiation and subsequent neuronal differentiation in Neuro2a cells, whereas srGAP3-IF-BAR and srGAP1-IF-BAR only induce filopodia formation. Although absence of the natural variants of the srGAP2 IF-BAR, SRGAP2B and SRGAP2C only in the human lineage encode truncated IF-BAR domains. Interestingly, forced ectopic expression of srGAP2B, has shown a similar activity with the full-length or “GAP-dead” srGAP2. It seems that at least partial IF-BAR domain of srGAP2 is required, not responsible for srGAP2 inhibiting neurite initiation and neuronal differentiation.

We demonstrate that srGAP1 and srGAP3 is a Rac1-specific GAP by RhoGAP pull down assay (Fig. 5; [20]). Although lack of RhoGAP activity, srGAP2 might play a role in neurite initiation in a srGAP3-dependent manner: srGAP2 or its GAP-dead mutant, srGAP2^R527A^ binds to srGAP3 and increases the Rac GAP activity of srGAP3 and subsequently inhibits neuronal differentiation. As srGAP1 possesses its own Rac GAP activity, srGAP1 and its GAP-dead mutants srGAP1^R542A^ exerts the inhibitory effect in two ways: (1) like srGAP2, srGAP1 also binds srGAP3, and/or (2) Like srGAP3, srGAP1 inhibits neuronal differentiation in a Rac1-dependent manner. It is very easy to understand why both srGAP1 and srGAP3, not srGAP2 inhibit neuronal differentiation of srGAP3-depleted Neuro2a cells. Absence of an effect of srGAP2 knockdown on neurite initiation is likely due to the presence of Slit-Robo1-srGAP3 pathway in Neuro2a cells.

SrGAPs possess SH3 domains, which bind to effectors [1], [17], i.e. Robo1 and some nucleation promoting factors [5], [33], [34], such as formin 1, WASP/WAVE, FMNL1 placing them in an ideal position to act at the interface between the plasma membrane and the actin cytoskeleton. The SH3 domains may be also involved in regulate IF-BAR-mediated membrane deformation and the “auto-inhibition” status of srGAPs [3], which will be used to explain that why the isolated IF-BAR domain and full length srGAP2 have a different effect on neuronal differentiation and neurite outgrowth.

To date, it has also been shown that RhoA [35], Rac1 [36] and several Rho GAPs localize to the nucleus or translocation between the nucleus and the cytosol, such as p190 RhoGAP [37], DLC1 [38] and srGAPs [9]. Our immunohistochemical analysis of srGAP2 and srGAP3 in Neuro2a cells reveals no distinguishable difference in endogenous subcellular localization, with both proteins producing punctuate staining throughout the cytoplasm, nucleus and protrusions (Fig. 2B; [20]. Importantly, neuronal srGAP2 and srGAP3 can be observed hetero-interaction in the subcellular nuclei fraction from P15 rat cereberal cortex by co-immunoprecipitation with srGAP3-3A1 and srGAP2-2A2 (Unpublished data). The findings of the present study do not exclude the possibility that nuclear srGAP2 and srGAP3 be involved in VPA-induced neuronal differentiation and neurite outgrowth in Neuro2a cells.

Acting as nuclear and cytoplasmic scaffold proteins, paralogous srGAPs proteins are likely to share other binding partners [17], [39], [40], such as YLPM1/ZAP3, Palladin, Gephyrin, DVL3, Lamellipodin, TNIK and Disc1. Among them, at least YLPM1/ZAP3, TNIK and Disc1 were reported to localize to nucleus [41]–[43]. The differences of srGAPs and their binding partners might also help to explain the differences between ours and others on observation of the srGAP2 activities in cortical neurons, N1E115 and Neuro2a. Of course, it will be very interesting to investigate the role of nuclear srGAP1, srGAP2 and srGAP3 in neuronal differentiation and neurite outgrowth.

Collectively, IF-BAR domains in srGAPs play important roles in the targeting of proteins (effectors and/or regulators) to specific regions within the plasma membrane where actin remodeling is needed (e.g., for formation of protrusions). More importantly, where srGAP3 or srGAP1 interacts with Robo1 through its SH3 domain to mediate Slit signaling. At these sites, srGAP3 or srGAP1 protein can control Rho GTPase activity, either by regulating the activation status of Rho GTPases, or by linking Rho GTPases to their upstream activators (e.g., Slit-Robo signaling) or to their downstream effectors (e.g., the actin machinery proteins such as WASP proteins and the Arp2/3 complex).

Inducing NB cells to differentiate is an important therapeutic approach that seems to be particularly promising for NBs [44]. Administration of VPA to NB cells such as Neuro2a, SHSY-5Y and BE (2)-C in vitro leads to proliferative arrest and neuronal differentiation [20], [45], [46]. Very recently, a whole-genome sequence analysis of 87 neuroblastomas reveals novel molecular defects in neurite genesis genes including a series of regulators of the Rac/Rho pathway, which frequently occur in high-risk tumours [47]. Our data presented that all srGAP1-3 inhibit VPA-induced neuronal differentiation in mouse Neuro2a cells. Among the srGAP family members, srGAP2 is highly expressed in mouse [20] and human [8], [19] NB cells. Thus, srGAP2 may also serve as a novel molecular target for human neuroblastoma therapy. More interestingly, it has been reported that SRGAP2B and SRGAP2C are partial duplications of srGAP2 gene only in the human lineage and encode truncated IF-BAR domains. The ancestral srGAP2 gene has been located at chromosome 1q32.1 and two duplicates located at 1q21.1 (SRGAP2B) and 1p12 (SRGAP2C) [8], [26]. SRGAP2C expressed in the developing and adult human brain shows that SRGAP2C is the most likely duplicate to encode a functional protein and blocks the action of ancestral SRGAP2 [8], [26]. It has recently become clear that, both a common deletion polymorphism at 1q21.1 [48] and CNVs in the 1q21.1 region [49] are associated with NB. Surprisingly, we found that SRGAP2B, not SRGAP2C inhibits VPA-induced neuronal differentiation, whereas srGAP2 IF-FBAR can facilitate VPA-induced neuronal differentiation of mouse Neuro2a cells. The data indicates a potential role of SRGAP2B in human NB. It seems even more confusing that srGAP2 IF-BAR, SRGAP2B and SRGAP2C have very distinct functions in filopodia formation, neuronal differentiation and spine maturation, although they maintain similar dimerization properties [8], [26] and lipid-binding activity (Figure S3). In the future, determination of crystal structures and identification of binding partners of srGAP2 IF-BAR, SRGAP2B and SRGAP2C will hopefully help to clarify these issues.

In summary, this study identifies synergistic interactions of srGAP1, srGAP2 and srGAP3 in regulation of neuronal differentiation and neurite outgrowth of VPA-induced Neuro2a cells. These findings may provide a potential mechanism of srGAPs in neuroblastoma tumorigenesis, neural development and neurodevelopmental diseases.

## Materials and Methods

### Ethics Statement

The investigation conforms to the Guide for the Care and Use of Laboratory Animals published by the US National Institutes of Health (NIH Publication No. 85-23, revised 1996), and the protocol was approved by the Animal Research Committee of Shanghai Jiao Tong University.

### Animals and brain tissue preparation

Postnatal (P) 15 days Sprague–Dawley (SD) rats were obtained from Shanghai Slac Laboratory Animal Company (Shanghai, CHINA). The pups were immobilized by anesthetized by injection of sodium pentobarbital (100–125 mg/kg of body weight (27 G×½″needle, volume ≤1% body weight). The cerebral cortices were dissected out on ice immediately after decapitation of the rat, frozen in liquid nitrogen, and stored at −70°C until lysis. The cortex tissue was lysed in 1∶20 ratio and sonicated on ice. Then the tissue homogenate was centrifuged at 12000 g for 15 min at 4°C. The supernatant was collected for RhoGAP Pull-Down Assay.

### The srGAP2-3 antibodies

Several antibodies against srGAP2 and srGAP3 were used in Western blot, immunofluorescence staining and immunoprecipitation (Figure S1A). Two peptide-affinity polyclonal antibodies against srGAP2, 2A1 (amino acids 193–205) and 2A2 (amino acids 873–890) were raised by Abmart (Shanghai, CHINA) [3], [8], [24]. Two polyclonal antibodies, srGAP3-3A1 (amino acids 870–882) [20], [24] and srGAP3-3A3 (amino acids 1088–1099) [20], [24] were homemade. The specificity of these purified antibodies was confirmed by Western blot and knockdown experiments.

### “GAP-dead” srGAP1^R542A^ and srGAP2^R527A^ constructs

The cDNAs that encoded an arginine→alanine substitution at amino acid residue 542 (R542A) in the full-length srGAP1 protein (BC053903 clone from PTGlab) and at residue 527 (R527A) in the full-length srGAP2 protein (KIAA0456 clone from Kazusa cDNA Insitute) were respectively generated using QuikChange Multi Site-Directed Mutagenesis Kit (Stratagene) according to the manufacturer's protocol. The following is the primer pairs used (5′ – 3′, base pair changes shown in bold).

srGAP1^R542A^-F:CTTCAGCATCAGGGGATTTTC**GC**AGTGTCTGGTTCCCAGG;

srGAP1^R542A^-R: CCTGGGAACCAGACACT**GC**GAAAATCCCCTGATGCTGAAG;

srGAP2^R527A^-F: CTACAGCATGAAGGAATTTTC**GC**GGTGTCAGGATCCCAGG;

srGAP2^R527A^-R: CCTGGGATCCTGACACC**GC**GAAAATTCCTTCATGCTGTAG.

### Cell culture, transfection and differentiation assay

HEK293FT cells (Invitrogen) were maintained in DMEM supplemented with 10% FBS, 100 U/mL penicillin and 100 µg/mL streptomycin (Invitrogen). The mouse NB cells, Neuro2a cells, were obtained from Institute of Biochemistry and Cell Biology, SIBS, CAS (Shanghai, China). They were cultured in DMEM containing 10% FBS, 1% non-essential amino acid (Invitrogen), 100 U/mL penicillin and 100 µg/mL streptomycin. HEK293FT cells or Neuro2a cells were transfected by using FuGENE HD Transfection Reagent (Roche) according to the manufacturer's instructions. Transient transfection conditions were optimized for maximum expression and minimal toxicity. Monitoring transfection efficiency by GFP is above 85%. To induce neuronal differentiation, Neuro2a cells (at about 20% confluence) were transferred to serum-free opti-MEM (Invitrogen) containing 1 mM VPA (Sigma) and allowed to extend neurites.

### Western blot

The Western blot assay had been previously described [20]. In brief, equal amounts of total protein (35 µg) were resolved and separated by 8% SDS–polyacrylamide gel electrophoresis (PAGE) and electro-blotted onto polyvinylidene difluoride membrane (PVDF; 0.2 µm, Roche). The membranes were treated with 1% blocking solution (w/v) in Tris-buffered saline (0.1 M Tris-HCl, pH 7.4, 0.1 M NaCl) for 1 hour and then incubated overnight at 4°C in 0.5% blocking solution with the primary antibody, anti-srGAP2 (2A1, 1∶1,000; 2A2, 1∶2,000); anti-srGAP3 (3A1, 1∶4,000; 3A3,1∶1,000); β-actin (Abmart,1∶1,000) and anti-GFP antibody (Abmart,1∶5,000). After incubation with POD-labeled secondary antibodies (Roche, 1∶12,500), the signals were revealed by BM Chemiluminescence Western Blotting kit (Roche). Prestained Protein Molecular Weight Marker (#SM0441, Fermentas) were used to determine the protein size. Densitometric quantitation was acquired in Gel Doc 1000 system and analyzed using Quantity One software (BioRad).

### Immunocytochemistry

Cells on Poly-L-Lysine-coated glass coverslips were fixed with 4% paraformaldehyde for 15 min at room temperature and then permeabilized with ice-cold methanol for 10 min. Cells were blocked by 10% normal donkey serum for 1 h, and then incubated at room temperature for 1 h with primary antibody diluted in antibody buffer (50 mM Tris-HCl, pH 7.4, 150 mM NaCl, 100 mM L-Lysine, 1% BSA and 0.04% azide). The following antibodies were used anti-srGAP2 (2A2, 1∶200) and anti-GAP-43 (1∶500). Phalloidin-TxR (Invitrogen, 1∶200) was used to label F-actin. After incubation with the primary antibodies, they were rinsed and incubated for 1 h at room temperature with Alexa Fluor-labeled secondary antibodies (Molecular Probes 1∶400–800). After washing, the coverslips were mounted with Glycerol/PBS containing 5 µg/mL Hoechst for nuclei staining. The labeled cells were observed under fluorescence microscope (BX-61) or Olympus Confocal Microscope (FV1000).

### Immunoprecipitation

The (co)-immunoprecipitations were obtained from double-transfected HEK293FT cells, lysed with nuclear lysis buffer (20 mM Tris-Cl pH 7.8, 125 mM NaCl, 5 mM MgCl2, 0.2 mM EDTA, 12% glycerol, 0.1% NP-40, complete protease inhibitors) 24–36 h after transfection. Incubations and washes were performed in the same buffer. 10% of lysis volume was collected prior to antibody incubations for input controls. The rest of the co-immunoprecipitation lysis was subjected to the immunoprecipitation antibodies (2 µg anti-GFP, Abmart, 2 µg 2A2, 2 µg 3A1,or 2 µg anti-IgG control antibody) bound to protein A/G beads (Santa Cruz), washed, and dissociated with SDS Loading Buffer at 95°C. Western blots were run as described before, using anti-GFP, anti-srGAP2 (2A2), or anti-srGAP3 (3A1 or 3A3) primary antibodies, and anti-mouse and anti-rabbit secondary antibodies described above.

### RNA Interference

A new shRNA against srGAP2 was constructed. According to the targeting sequences of srGAP2, a pair of oligonucleotide encoding shRNA was designed using BLOCK-iT™ RNAi Designer (Invitrogen). The oligonucleotides were annealed and cloned into the pcDNA6.2™-GW/EmGFP vector (Invitrogen) to generate J24 shRNA expressing plasmid according to the instruction of the BLOCK-iT™ Lentiviral Pol II miR RNAi Expression Vector Kits (Invitrogen). The sequence of J24 shRNA are shown as below:

5′-TGCTGTAAAGAGTCTTCCTGAGTCCTGTTTTGGCCACTGACTGACAGG ACTCAAAGACTCTTTA-3′ (Sense) and 5′-CCTGTAAAGAGTCTTTGAGTCCTGTCAGTCAGTGGCCAAAACAGGAC TCAGGAAGACTCTTTAC-3′ (Anti-sense).

### RhoGAP Pull-Down Assay

The RhoGAP pull-down assay had been previously described [20], [25]. Briefly, plasmids were transformed into the E. coli strain BL21 (DE3). Protein production was induced with 100 µM Isopropyl β-D-1-thiogalactopyranoside (TAKARA) at 16°C for 18 h. GST-fusion proteins were purified in batch on Glutathione-Agarose (Sigma). Cells and brain tissues were lyzed in 20 mM HEPES (pH 7.5), 150 mM NaCl, 5 mM MgCl2, 1% TritonX-100, and 1 mM dithiothreitol with protease inhibitors. About 3,000 µg of protein lysates were incubated with 60 µg of purified Rho GTPase (CA) bound to glutathione-agarose for 2 h at 4°C, followed by washing three times with lysis buffer. Precipitated proteins were then solubilized in sample buffer and analyzed by Western blot.

### Statistical analysis

For assessing differentiation, Neuro2a cells grown in 35-mm dishes were treated with opti-MEM containing VPA for 24 h. Cells were viewed with an inverted phase contrast microscope (Nikon Instruments Inc., Melville, NY) and photographed. Three –seven images were taken from randomly selected areas in each of at least four wells. To quantify neurite initiation, the percentage of neurite bearing cells was calculated for each image as the ratio of neurite bearing cells to the total number of cells in four categories (as shown in Fig. 1B and G). Cells with neurite processes longer than two cell bodies were considered to be those differentiated. Each group was evaluated by counting about 150–500 cells. Assessment of neurite outgrowth was performed by counting about 30–50 cells per condition. Neurite length and neurite number were quantified using Image Pro-Plus software. In all analysis, the data represents Mean ± S.D. of 3–6 independent experiments. For comparison, statistical significance was tested by paired *t*-test or one-way ANOVA.

## Supporting Information

Figure S1 The recognition sites for different antibodies against srGAP2 and srGAP3. A. Schematic representation of the structure of full-length human srGAP1-3 proteins, which mainly contain an IF-BAR domain, a RhoGAP domain and a SH3 domain. The red font represents homology percentage of the IF-BAR, RhoGAP and SH3 domains from srGAP1 and srGAP2 compared to srGAP3. The arrowheads indicate the different target regions of srGAP2-3 specific polyclonal antibodies. B. HEK293FT cells were transfected with GFP mock vector or GFP-tagged full-length srGAP2. Cell lysates were detected by Western blot with GFP, 2A1 and 2A2 antibodies respectively. C. Sequence alignment of the srGAP-RhoGAP domains of srGAP1, srGAP2 and srGAP3 by ClustalW. The conserved Arginine finger (R) is boxed in red.
(TIF)

Figure S2 Knockdown efficacy of J24 shRNA against srGAP2. A–B. Control shRNA and J24 shRNA were transfected to HEK293FT (A) and Neuro2a cells (B) respectively, and then blotted with srGAP2 antibody. β-actin was selected as a loading control.
(TIF)

Figure S3 SRGAP2B and SRGAP2C lose filopodia-inducing activity. A. Representative immunoblot analysis of SRGAP2B/SRGAP2C and lipid interactions. Purified GST fusion proteins, GST-SRGAP2B and GST-SRGAP2C were incubated with membranes containing an array of membrane lipid spots as shown in Figure, and then detected by Western blot with GFP antibody. Phosphatidic acid (PA); Phosphatidylinositol (4, 5)-bisphosphate (PIP2); Phosphatidylinositol 3,4,5-trisphosphate (PIP3); PtdIns, Phosphatidylinositol; DAG, diacylglycerol; Sulfatide, 3-sulfogalactosylceramide. B. Two human duplications of SRGAP2 gene, SRGAP2B and SRGAP2C with GFP tag were transfected into HEK293FT cells, and immunostained with F-actin antibody. The arrowheads indicate the cell protrusions. C. GFP-tagged SRGAP2B and SRGAP2C were transfected into Neuro2a cells. The F-actin of undifferentiated (UD) or differentiated (VPA) cells were labeled with Texas Red-X phalloidin, respectively. Bar = 20 µm.
(TIF)

Methods S1 Lipid array overlays.
(DOC)

Results S1 SRGAP2B and SRGAP2C bind to negatively charged phospholipids.
(DOC)
