# Supplementary figures and images for: The Inverse F-BAR Domain Protein srGAP2 Acts through srGAP3 to Modulate Neuronal Differentiation and Neurite Outgrowth of Mouse Neuroblastoma Cells
Source: PLoS One. 2013 Mar 7;8(3):e57865. doi: 10.1371/journal.pone.0057865 (PMC3591447; doi:10.1371/journal.pone.0057865)

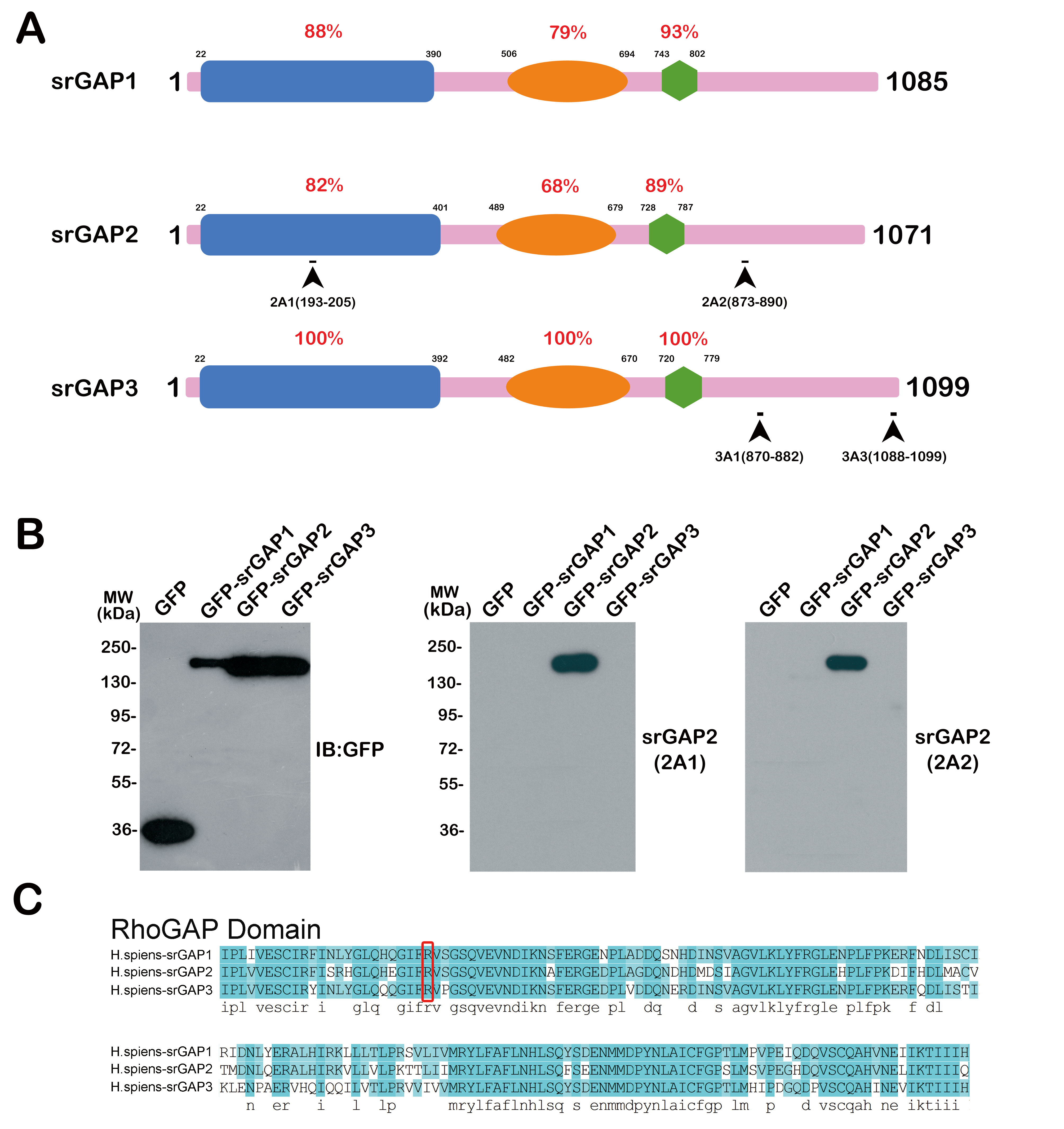

Supplement: Figure S1 — The recognition sites for different antibodies against srGAP2 and srGAP3. A. Schematic representation of the structure of full-length human srGAP1-3 proteins, which mainly contain an IF-BAR domain, a RhoGAP domain and a SH3 domain. The red font represents homology percentage of the IF-BAR, RhoGAP and SH3 domains from srGAP1 and srGAP2 compared to srGAP3. The arrowheads indicate the different target regions of srGAP2-3 specific polyclonal antibodies. B. HEK293FT cells were transfected with GFP mock vector or GFP-tagged full-length srGAP2. Cell lysates were detected by Western blot with GFP, 2A1 and 2A2 antibodies respectively. C. Sequence alignment of the srGAP-RhoGAP domains of srGAP1, srGAP2 and srGAP3 by ClustalW. The conserved Arginine finger (R) is boxed in red. (TIF) [file pone.0057865.s001.tif]

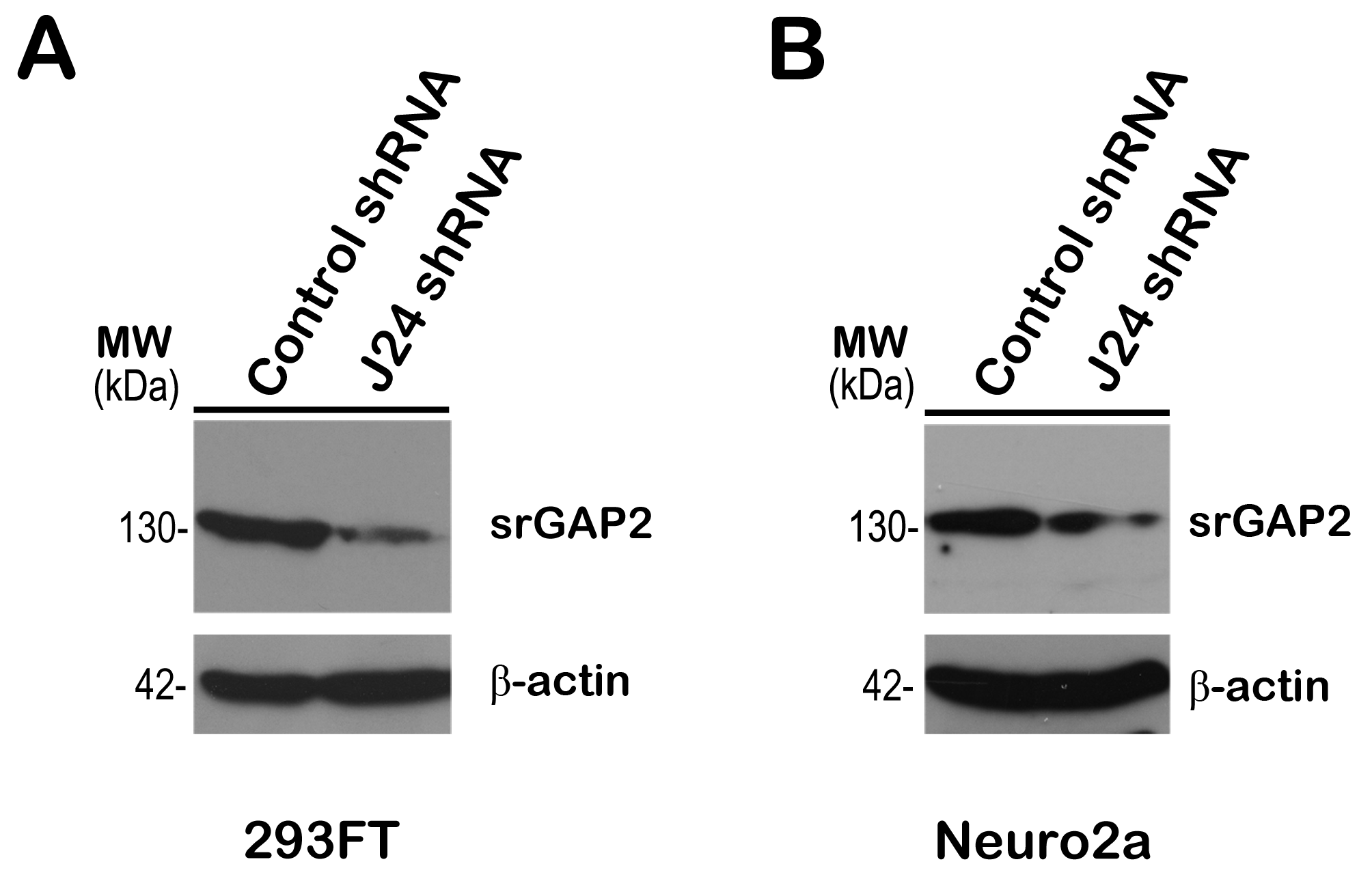

Supplement: Figure S2 — Knockdown efficacy of J24 shRNA against srGAP2. A–B. Control shRNA and J24 shRNA were transfected to HEK293FT (A) and Neuro2a cells (B) respectively, and then blotted with srGAP2 antibody. β-actin was selected as a loading control. (TIF) [file pone.0057865.s002.tif]

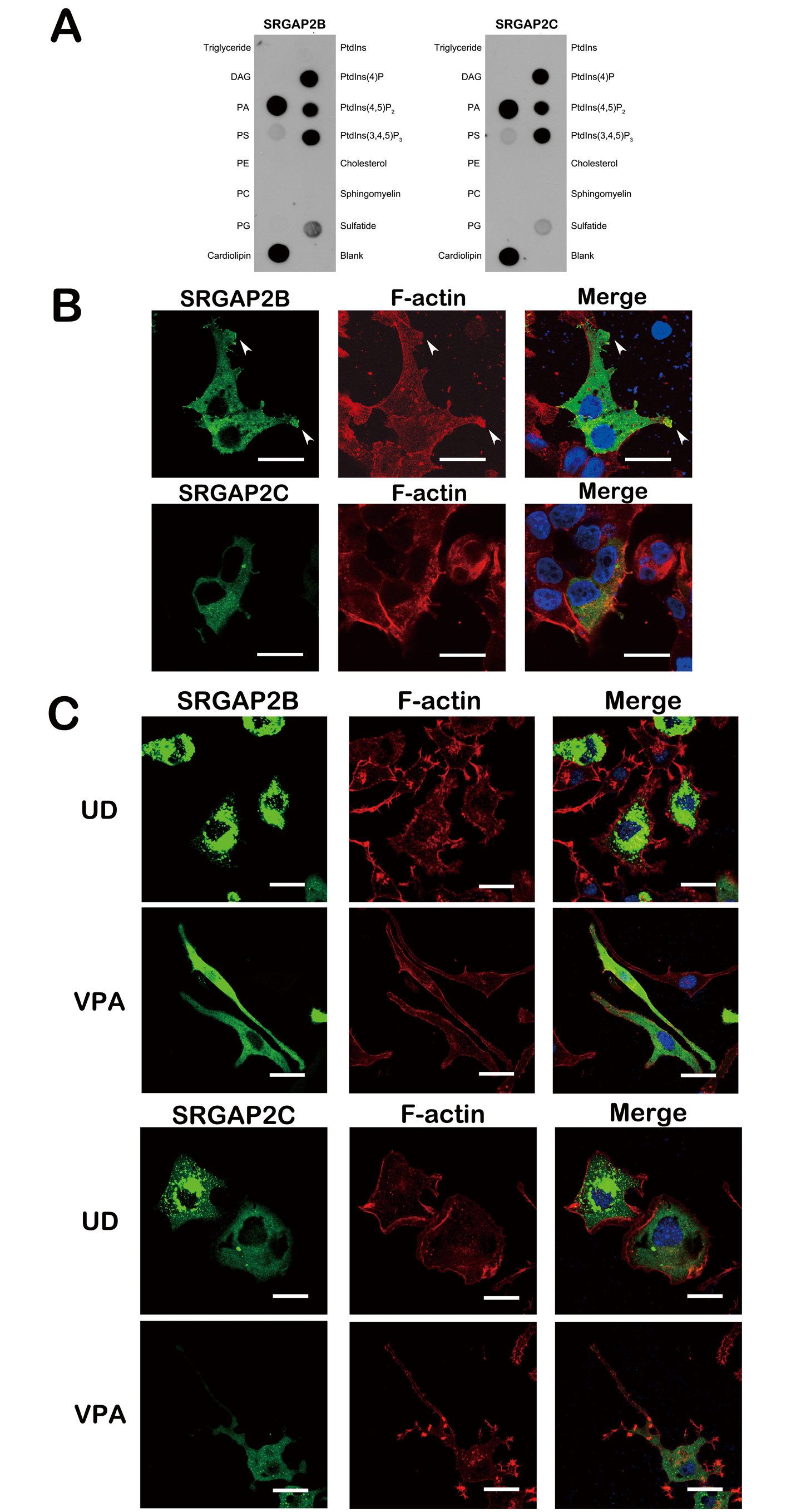

Supplement: Figure S3 — SRGAP2B and SRGAP2C lose filopodia-inducing activity. A. Representative immunoblot analysis of SRGAP2B/SRGAP2C and lipid interactions. Purified GST fusion proteins, GST-SRGAP2B and GST-SRGAP2C were incubated with membranes containing an array of membrane lipid spots as shown in Figure, and then detected by Western blot with GFP antibody. Phosphatidic acid (PA); Phosphatidylinositol (4, 5)-bisphosphate (PIP2); Phosphatidylinositol 3,4,5-trisphosphate (PIP3); PtdIns, Phosphatidylinositol; DAG, diacylglycerol; Sulfatide, 3-sulfogalactosylceramide. B. Two human duplications of SRGAP2 gene, SRGAP2B and SRGAP2C with GFP tag were transfected into HEK293FT cells, and immunostained with F-actin antibody. The arrowheads indicate the cell protrusions. C. GFP-tagged SRGAP2B and SRGAP2C were transfected into Neuro2a cells. The F-actin of undifferentiated (UD) or differentiated (VPA) cells were labeled with Texas Red-X phalloidin, respectively. Bar = 20 µm. (TIF) [file pone.0057865.s003.tif]
