# Supplementary material for: The Inverse F-BAR Domain Protein srGAP2 Acts through srGAP3 to Modulate Neuronal Differentiation and Neurite Outgrowth of Mouse Neuroblastoma Cells
Source: PLoS One. 2013 Mar 7;8(3):e57865. doi: 10.1371/journal.pone.0057865 (PMC3591447; doi:10.1371/journal.pone.0057865)
Supplement: Methods S1 — Lipid array overlays. (DOC) [file pone.0057865.s004.doc]

**Supplementary Methods**

**Lipid array overlays**

The isolated IF-BAR domain of srGAPs, SRGAP2B and SRGAP2C, were all expressed as N-terminal GST fusions in bacteria (BL-21). Proteins were purified using Glutathione-Agarose (Sigma).

Lipid array overlays were performed according to the manufacturer’s instructions (Echelon). Membranes prespotted with lipids were blocked in 1% Blocking solution (Roche) in TBS with 0.2% Tween 20 at room temperature for 1 h. Membranes were then incubated with 0.5 μg/ml the purified GST fusion proteins overnight at 4°C and then Western blotted for GST (1:2,000). The membrane lipids spot shown in Figure S3 as the follows：Phosphatidic acid (PA); Phosphatidylinositol (4, 5)-bisphosphate (PIP2); Phosphatidylinositol 3,4,5-trisphosphate (PIP3); PtdIns, Phosphatidylinositol; DAG, diacylglycerol; Sulfatide, 3-sulfogalactosylceramide.
