# Supplementary material for: The Inverse F-BAR Domain Protein srGAP2 Acts through srGAP3 to Modulate Neuronal Differentiation and Neurite Outgrowth of Mouse Neuroblastoma Cells
Source: PLoS One. 2013 Mar 7;8(3):e57865. doi: 10.1371/journal.pone.0057865 (PMC3591447; doi:10.1371/journal.pone.0057865)
Supplement: Results S1 — SRGAP2B and SRGAP2C bind to negatively charged phospholipids. (DOC) [file pone.0057865.s005.doc]

**Supplementary Results**

***SRGAP2B and SRGAP2C bind to negatively charged phospholipids***

Previous studies had shown that IF-BAR domain recombinant proteins of srGAP2 and srGAP3 can bind to negatively charged phospholipids using lipid array overlays assay. We also extended the assay of SRGAP2B and SRGAP2C, products of two human duplications of SRGAP2 gene, encoding a truncated IF-BAR domain . Purified GST fusion proteins of SRGAP2B and SRGAP2C were screened for its ability to directly bind lipids by overlay onto membranes containing an array of membrane lipid spots. Both the recombinant proteins bind several lipids, including PA, PIP2, PIP3, and cardiolipin. Importantly, binding was not observed with PS, another negatively charged lipid, which confirmed the specificity of the binding assay (Figure S3A).
